# Supplementary material for: Nasal Microbiota Modifies the Effects of Particulate Air Pollution on Plasma Extracellular Vesicles
Source: Int J Environ Res Public Health. 2020 Jan 17;17(2):611. doi: 10.3390/ijerph17020611 (PMC7013854; doi:10.3390/ijerph17020611)
Supplement: Supplementary file 1 [file ijerph-17-00611-s001.pdf]

Table S1: Association between PM exposure and different classes of EV, stratifying subjects by their *Moraxell*a genus relative abundance into Mor- (≤25%) and Mor+ (>25%) group. All the multivariable linear regression models were adjusted for age, gender, smoking behavior (Never smoker, Former smoker, Current smoker), and BMI.

		**MOR- (n = 30)**			**MOR+ (n = 10)**			***p for interaction***
		**GMR**	**(95% CI)**	***p***	**GMR**	**(95% CI)**	***p***	
**$PM_{2.5}$ exposure**								
**EV count**[A] (10⁶/ml PL)	Total EV	1.92	(1.12-3.30)	0.02	0.78	(0.40-1.52)	0.36	0.05
	Exosomes	1.11	(0.35-3.54)	0.85	0.14	(0.001-7.458)	0.26	0.02
**EV subtypes**[B] (10^3/ ml PL)	CD61+ (platelets)	2.98	(0.51-17.3)	0.21	0.81	(0.004-183)	0.92	0.58
	CD66+ (neutrophils)	2.63	(0.51-13.5)	0.23	0.004	(0.001-0.14)	0.01	0.17
	EpCAM+ (epithelium)	2.68	(0.91-7.93)	0.07	0.005	(0.001-0.11)	0.01	0.10
	CD105+ (endothelium)	4.39	(1.27-15.1)	0.02	0.1	(0.002-4.30)	0.17	0.47
	CD14+ (macr./monoc.)	5.34	(1.09-26.1)	0.04	0.04	(0.003-0.49)	0.02	0.24
**PM_{10} exposure**								
**EV count**[A] (10^6/ml PL)	Total EV	1.94	(1.09-3.43)	0.02	0.81	(0.37-1.74)	0.49	0.04
	Exosomes	1.23	(0.36-4.12)	0.73	0.1	(0.001-7.45)	0.21	0.02
**EV subtypes**[B] (10^3/ ml PL)	CD61+ (platelets)	3.54	(0.57-22.1)	0.17	0.48	(0.001-164.7)	0.75	0.78
	CD66+ (neutrophils)	2.89	(0.53-15.9)	0.21	0.002	(0.00007-0.05)	0.01	0.10
	EpCAM+ (epithelium)	3.27	(1.08-9.91)	0.04	0.003	(0.00009-0.11)	0.01	0.04
	CD105+ (endothelium)	5.38	(1.51-19.1)	0.01	0.06	(0.001-2.71)	0.11	0.26
	CD14+ (macr./monoc.)	5.42	(1.02-28.9)	0.05	0.02	(0.003-8.83)	0.01	0.16

GMR, Geometric Mean Ratio

[A] Counts of EV fractions obtained by Nanosight analysis

[B] Counts of EV fractions obtained by Flow-cytometry
